# Acceptability, feasibility, and effectiveness of WE-SURF™: a virtual supervised group-based fall prevention exercise program among older adults

**DOI:** 10.1007/s40520-024-02759-x

**Published:** 2024-06-05

**Authors:** Janet Bong May Ing, Maw Pin Tan, Julie Whitney, Ing Khieng Tiong, Devinder Kaur Ajit Singh

**Affiliations:** 1 Physiotherapy Programme, Centre for Healthy Ageing and Wellness, Faculty of Health Sciences, Universiti Kebangsaan Malaysia, Kuala Lumpur, Malaysia; 2 Physiotherapy Unit, Sarawak Heart Centre, Sarawak Health Department, Ministry of Health Malaysia, Sarawak, Malaysia; 3 Division of Geriatric Medicine, Department of Medicine, Faculty of Medicine, University of Malaya, Kuala Lumpur, Malaysia; 4 King’s College London, London, UK; 5 Geriatric Unit, Sarawak Heart Centre, Sarawak Health Department, Ministry of Health Malaysia, Sarawak, Malaysia

**Keywords:** Virtual group-based exercise, Fall prevention, Older adults

## Abstract

**Abstract:**

Conducted physically, supervised group-based falls prevention exercise programs have demonstrated effectiveness in reducing the risk of falls among older adults. In this study, we aimed to assess the acceptability, feasibility, and effectiveness of a virtual supervised group-based falls prevention exercise program (WE-SURF™) for community-dwelling older adults at risk of falls.

**Method:**

A preliminary study utilizing virtual discussions was conducted to assess the acceptability of the program among six older adults. Effectiveness was evaluated in a randomized controlled feasibility study design, comprising 52 participants (mean age: 66.54; SD: 5.16), divided into experimental (n = 26) and control (n = 26) groups. The experimental group engaged in a 6-month WE-SURF™ program, while the control group received standard care along with a fall’s prevention education session. Feasibility of the intervention was measured using attendance records, engagement rates from recorded videos, dropouts, attrition reasons, and adverse events.

**Results:**

Preliminary findings suggested that WE-SURF™ was acceptable, with further refinements. The study revealed significant intervention effects on timed up and go (TUG) (η2p:0.08; p < 0.05), single leg stance (SLS) (η2p:0.10; p < 0.05), and lower limb muscle strength (η2p:0.09; p < 0.05) tests. No adverse events occurred during the program sessions, and both attendance and engagement rates were high (> 80% and 8/10, respectively) with minimal dropouts (4%). The WE-SURF™ program demonstrated effectiveness in reducing the risk of falls while enhancing muscle strength and balance.

**Conclusion:**

In conclusion, WE-SURF™ was demonstrated to be an acceptable, feasible, and effective virtual supervised group-based exercise program for fall prevention in community-dwelling older adults at risk of falls. With positive outcomes and favourable participant engagement, WE-SURF™ holds the potential for wider implementation. Further research and scaling-up efforts are recommended to explore its broader applicability.

(Registration number: ACTRN 12621001620819).

## Introduction

Globally, the prevalence of falls among older adults is expected to rise due to factors such as a rapidly aging population, polypharmacy, multimorbidity (including frailty), and cognitive impairment [2]. At least one third of older adults aged 65 years and above worldwide have been reported to experience at least one fall each year [3]. Similarly, fall prevalence in Malaysian older adults’ ranges from 4.2% to 47% depending on settings [4–9] with an incidence rate of occasional and recurrent falls of 8.47 and 3.21 per 100 person-years respectively [10].

The consequences of falls are debilitating and leads to increased personal, societal and economic burden. An estimated 17 million years of life lost (YLLs), 20 million years lived with disability (YLDs) and 36 million disability-adjusted life years (DALYs) was reported in the Global Burden of Disease 2017 study due to falls and associated adverse outcomes [11]. There is a need to implement falls risk reduction initiatives on a broader scale for older adults, as indicated in the World Falls Guidelines [12].

Although, falls are considered multifactorial in nature, muscle weakness and poor balance are among the main risk factors for increased falls among older adults [7, 13–15]. In addition, compared to their counterparts in Western countries, the physical activity level of community-dwelling older people in Southeast Asia is generally considered inadequate [16]. The prevalence of low physical activity among Malaysian older adults has been reported as 21% [17]. In contrast, the overall prevalence of physical inactivity in older adults across 16 European countries was found to be 12.5% [18].

A structured and planned exercise program may be able to counteract fall risk factors such as muscle weakness and impaired balance. A meta-analysis with 44 studies found that regular exercise that challenges balance is the most effective method to prevent falls or fall injuries [19]. A subsequent Cochrane review found that multicomponent exercise is effective in reducing rate of falls by 34% (95% CI 12% to 15%) [20].

The World Falls Guidelines recommends exercise that challenges balance and includes strengthening and functional components undertaken at least three times a week, progressed in intensity for at least 12 weeks and continued longer for greater effects to prevent falls in community dwelling older adults [2]. Other effective falls prevention programs range from simple falls prevention education [21, 22] to more challenging exercises such as square stepping exercises [23, 24] and backward chaining exercises [25]. Furthermore, it is crucial to design exercises that are suitable for the targeted group to ensure both adherence to and the effectiveness of exercise programs [1, 26–29].

Despite physical exercise and activity having sufficient evidence for prevention and management of falls and falls related issues in older adults, the uptake of both remains low, more so in Malaysia [12, 30]. There are also limited opportunities for older adults to engage in evidence based and supervised exercise programs, particularly for those with low to intermediate falls risk. Moreover, older adults with falls who have been managed in healthcare facilities may not be adequately self-efficient for compliance in self- management, especially with regards to maintaining regular participation in physical exercise programs [31, 32].

Recent reports indicate that virtual group-based exercise programs are convenient and feasible for Malaysian older adults [33]. Additionally, it’s noteworthy that more than 80% of healthy Malaysian older adults are likely to use digital technology [34]. Furthermore, online falls prevention interventions, are shown to have a significant improvement in fall risk, balance, and fall efficacy [35]. To address the issues of poor uptake and adherence of evidence-based fall prevention exercise among Malaysian older adults we tested the feasibility, acceptability and effectiveness of a virtual fall prevention exercise group.

## Methodology

### Study design

This was a randomized controlled feasibility trial conducted between March 2021 and October 2021. Human research ethics approval was obtained through the Medical Research and Ethics Committee of the University (Universiti Kebangsaan Malaysia (JEP-2020-611) and the Medical Research and Ethics Committee, Ministry of Health, Malaysia (NMRR-20–2289-56760). The trial was registered with the Australian New Zealand Clinical Trials Registry (Registration number: ACTRN 12621001620819).

A preliminary study was carried out to assess the acceptability of the program among six participants (mean age: 66.2 years, 61–76 years) and to determine optimal delivery strategies. Virtual discussions were conducted with the participants using semi-structured questions after two trial sessions of the level 1 virtual exercise program. The moderator (one of the researchers) conducted the discussions, assisted by one co-moderator (a physiotherapist), who was not involved in the intervention design or delivery.

The sample size calculation for the trial was based on the G*power 3.1 statistical power analysis program [36]. The power analysis indicated that a total sample of 42 participants was required to detect a significant difference between two groups, with an effect size of 0.40 (35), using the time up and go test (TUG) as the primary outcome indicative of the risk of fall. While α represents the significance level (p < 0.05), and 1-β err prob denotes the power, set at 0.80. A 20% dropout rate was estimated and thus a total sample size of 52 participants (26 participants per group) was considered adequate for this study.

### Participants

Participants were recruited via poster and word of mouth, advertised at government clinics, hospitals, community groups such as local churches, mayoral offices, senior activity centres (PAWE), and volunteers from local non-governmental organizations (Sarawak Gerontology and Geriatrics Association, Women’s Institute Kuching). Fifty-two adults aged 60 years and older with risk of falls, measured using the timed up and go (TUG) test with a cut-off value of 11.2 s and below, living in the urban city area around Kuching, Sarawak participated in this study. Kuching, Sarawak is located in the north western region of the island of Borneo in Malaysia. Exclusion criteria included those who were unable to complete an hour’s exercise program, have acute or chronic conditions, including severe musculoskeletal pain, recent history of fracture within the last 6 months, cardiovascular diseases like stroke with hemiplegia, unstable angina. neurological diseases with progressive weakness, postural hypotension and, vestibular problems. Those with uncorrected visual issues, cognitive deficits (assessed using Montreal Cognitive Assessment (MoCA) test with score less than 20) [37] and those participating in other exercise programs in the previous 6 weeks or illiterate were also excluded.

### The intervention

Development of WE-SURF ™ was adapted from available evidence on falls prevention programs [22, 23, 25, 38–40] to suit the local older population. WE- SURF™ stands for “Warga Emas—Steady, UpRight and Firm exercise. “Warga Emas” is the Malaysian language term for older adults. “Steady, UpRight and Firm” represents our objectives to optimize fall risk, balance and muscle strength.

WE-SURF™ is a 24-week virtual supervised group-based exercise program of three levels progressive exercise. It included one session of falls prevention program education that covered topics such as an overview of falls, consequences of falls in older adults, fall risk factors, fall prevention strategies, and how to get up after a fall. The preparation of an online class included discussion and question–answer sessions on the set up of the tools for virtual exercises and a trial session demonstration. There was a total of 48 online exercise sessions. Exercises included warm up, resistance exercise using strap on weights, balance training, square-stepping, cool down and backwards chaining to train for getting up from floor. WE-SURF TM was delivered online via Zoom™ and supervised by a physiotherapist with 10 years’ experience. Each WE-SURF™ duration ranged from 75–90 min with group-sessions including a maximum of seven older persons. The virtual session was conducted twice per week for 6 months. In the first online education session, participants were requested to test their smart mobiles, tablets or computers and any technical issues were solved with the help of the therapist who was online and family members at home. This included placement of the smart device for better viewability, home space utilization and safety. The overview of WE-SURF™ program is as depicted in the Template for Intervention Description and Replication (TIDieR) (Table 1).

**Table 1:** The Template for Intervention Description and Replication (TIDieR) for WE-SURF ™ program

TIDier Checklist						
Exercise type	Intervention location	Intervention provider	Procedure materials	When how much	Tailoring	Fidelity
Multicomponent exercises: 1. Warm up -aerobic exercise 2. Resistance exercise -upper limb to lower limb 3. Challenge balance training -static balance to dynamic balance 4. Square stepping exercise 5. Backward chaining training 6. Cool-down	Online video conferencing via the Zoom platform	Physiotherapist	-Oldies song and music -Ankle and wrist adjustable weight -Firm Chair -Pillow -Yoga mat -Square-stepping mat (25 m × 10 m) -Small ball -Cup with holder -Towel	-Twice per week, 24 weeks -75 to 80 min per session -Total 48 sessions -Progress from level 1 Light (9–12) RPE Level 2 Somewhat hard (11–15) RPE Level 3 Very hard (14–17) RPE	-Those with hip or knee pain and unable to kneel down -Reduce intensity and increase thickness of mat with pillow support	Attendance rate Attrition reasons Engagement rate Dropout rate Adherence to program components

### Control group

Participants in the control group attended one session of virtual fall prevention education session and continued their usual care or activities routine without any restrictions or changes until the end of the study.

### Baseline measures

#### Sociodemographic information

Sociodemographic information was obtained from all participants through a standardized data collection document. Details obtained included gender, race, ethnicity, education level, marital status, living arrangements, employment status, medical history, alcohol intake, smoking status, history of falls within the past 12 months and comorbidities. Modified Baecke physical activity score, cognitive deficits based on MoCA test scale and Geriatric Depression score (GDS) were also obtained.

The modified Baecke physical activity score was used to assess the level of physical activity. It comprises 19-items within categories of household activities, sports activities and leisure time activities rated using a five-point Likert scale. The test–retest validity was reported to be good for culturally diverse populations [41] and older adults [42].

A short-form GDS was used to indicate depression level for the participants. Scores of less than 4 were considered normal; 5–8 considered mild depression; 9–11 indicated moderate depression; and more than 12 indicated severe depression. While validated GDS-15 consisted of two domains; depression and psychosocial activities, only the overall score was considered [43]. The reliability was considered good with reported overall value for Cronbach’s alpha: 0.890.

### Acceptability of the exercise intervention

Acceptability was ascertained in the preliminary study with virtual discussion to explore participants experience and perceptions towards the WE-SURF™ exercise program. Semi structured questions were used to conduct one discussion interview via Zoom ™. Participants were also asked to rate the level of acceptability after two sessions of trials virtual exercise program on 1–10 Likert scale with 10 being completely acceptable.

### Feasibility of the exercise intervention

The feasibility of the intervention measured using attendance records, engagement rate from the recorded videos, dropouts, attrition reasons, and adverse events. Attendance of each participant in every session was recorded; dropouts and attrition reasons or reason of absence for each session were also recorded. The engagement rate was determined based on three randomly chosen recorded videos for each level and assessed by another blinded assessor (a physiotherapist not involved in the study). Each participant was rated from 0 to 10 (none to full engagement) based on their engagement throughout the session for each level. Participants were requested to report any adverse events, such as musculoskeletal pain, falls, or hospitalization, to the instructor during the program and the 6-month assessment.

### Primary outcome measurements

#### Time up and go test (TUG)

The TUG test has a high test–retest reliability (ICC = 0.92–0.0.99) in older adults [44]. From a sitting position on an armrest chair, each participant was requested to stand, walk three metres at a comfortable pace, turn, and walk back to the chair and sit down. The time taken was recorded in seconds, from the time the participant began to stand until the participant sat back down. Participants performed this test three times; average scores recorded in seconds were used in the analysis.

#### Single leg stand test (SLS)

The SLS test is a reliable clinical test which assessed the static balance [45]. The time from when participant lifted one leg and stands on one foot without arm support until the lifted leg touched the ground was recorded in seconds. Each participant was required to perform this test three times with the longest performance recorded as the final score.

#### Dominant handgrip strength (HGS)

The dominant HGS is a valid and reliable measurement tool to determine upper limb muscle strength. The test–retest reliability for the dominant HGS test was (ICC = 0.97, p = 0.01) [46] Participants were asked to use a standard dynamometer (Patterson Medical, UK) to apply their maximum grip strength in a sitting position with the arm adducted, the forearm unsupported, the elbow flexed at 90 degrees, and the wrist in a neutral position. Three tests were performed with the highest value recorded as the participant’s grip strength.

#### Five times sit to stand test (5TSTS)

The 5TSTS test is a clinical test used to measure lower extremity strength. It has high test–retest reliability (ICC = 0.81) [47]. Participant stood up from the chair and sat back down for a total of five repetitions. Time recording started from the moment standing was initiated and ended after five repetitions of the STS tasks were completed.

### Secondary outcome measurements

#### Quality of life (EQ-5D)

The EQ-5D has been widely translated and its validity within the Malaysian population has been established [48]. We used both the Malay and English versions of the EQ-5D. The Visual Analogue Scale (VAS) was used in this questionnaire for participant report of perceived health status which ranged from 0 (worst health status) to 100 (best possible health status). The EQ-5D questionnaire has an acceptable test–retest reliability value ICC = 0.79 [49].

#### Short fall efficacy scale-international (FES-I)

The short FES-I consists of seven items [50] which measures the level of fear of falling (FoF) during the physical and social activities of daily life. The FES-I has a high test–retest reliability (ICC = 0.93) and correlates with both the level of anxiety and getting up from a fall. Evidence shows it is reliable and valid for older adults [51]. The short FES-I is considered preferable in the Malaysian cultural population compared to the original 16-item version of the FES-I considering the longer version comprises items activities of daily living not routinely performed by the Malaysian older adults [52]. A fall diary was given to the participants or their carers to record any fall episodes.

### Randomisation and blinding

The assessor who assessed the pre-post measures, a physiotherapist, was blinded to the groups. Since the recruitment process involved one-to-one screening, block randomisation was carried out based on the recruitment batches [53]. Each batch consisted of 12 to 14 participants. Average time from baseline assessment to randomisation was within 1 week. Participants were randomised into the experimental or control groups via block randomisation. The randomisation of the participants was conducted by a research assistant, who was not involved in the design, delivery, or assessment of the intervention or intervention outcomes. The randomisation used a computer-generated random number table from the free online randomisation website www.randomizer.org. Four blocks of randomisation were carried out individually based on availability of the participants.

### Data analysis

For the preliminary study, qualitative data was analysed thematically. The discussion was audio-recorded, transcribed, and coded using the thematic analysis method for qualitative data analysis. The complete transcripts were sent to participants to ensure trustworthiness and data familiarity to ensure rigorous data analysis [54].

All statistical analysis was carried out using the Statistical Package for Social Sciences (SPSS) software, version 24.0. An alpha level of (0.05) was considered for all the statistical tests. For the descriptive analysis, statistics on clinical parameters and participants’ characteristics were reported by presenting frequencies via percentages or means with standard deviations. The experimental and control groups were compared using the chi-square test for dichotomous variables, while the t-test was employed for continuous variables. The results of the randomized trial were analysed using repeated measures analysis of variance to assess within, between, and interaction effects for the primary outcome, which included the risk of fall (TUG test), balance, muscle strength assessment, and secondary outcome measurements.

In the main analyses, participants were included in the groups as they were randomized and the intention-to-treat principle was used. In the analyses of rates and dropouts, censored observations were considered with reasons for dropout reported. Additional analyses were conducted where missing data were imputed using baseline assessment data.

## Results

### Acceptability

One researcher at a public hospital conducted screening and identified 6 out of 20 older adults as eligible participants. A total of 6 participants were enrolled in the preliminary study, with a mean age (SD): 66.17 ± 4.67 years. Three themes providing participant views emerged from the discussion (Table 2).

**Table 2:** The themes, subthemes, and quotes from the preliminary study

Themes	Subthemes	Quotes from participants
Experience of getting involved in virtual exercise	New experience	***P1****: I never had joined any virtual or online exercise program, this is first time to me*
	Learning	***P2:**** Some parts of exercise are new to me; I learn how to do it* ***P4****: This virtual exercise program is a good opportunity to do exercise at home. We can learn about it* ***P5****: I think instructed program can [ help me] learn better than exercise alone with video*
Exercise program’s characteristic	Intensity of the exercise	***P6****: 2 session per week is ideal to me, if three times per week, I can’t accept but is hard to commit* ***P3:**** For the time being, [it] is better do [ the session] …instructed and supervise [d by the] instructor, we can learn now and proceed without supervise in the future. Two days per week, around 1 h is good so that [is] enough time for us to do it. Too short time might be rush[ed] to us too. I prefer less than 10 participants in one session, and we just follow how long the exercise program is* ***P2****: Two to three sessions per week should be fine, and around 1 h plus per session is good. Because sometimes we need to spare time for others to ready themselves and set up their space. If [the time is] too short, will be very rush. I don’t think is suffiecient to us too. This program should be 1 year duration, then only [it is] enough for us*
	Method of delivery and exericise components	***P1***: *I prefer virtual because I can’t drive, [it is] more convenient and can do at home. I must depend on somebody, is not easy* ***P2****: I still prefer face-to-face because instructor can correct us on the spot. The interaction between each other is more “real”* ***P3***: *It is good to do exercise at home, [it is] convenient as I don’t have transport and [it] is time-consuming to go down [is] conducted at community centre* ***P5:**** Compare virtual and face-to-face exercise program, face-to-face program is better but online is not bad too* ***P6:**** I love the aerobic exercise, [it was] more fun and I sweat more during aerobic exercise. I can perform kneeling but [it is] difficult to me sit down on the floor. I feel heavy, [and it is] difficult to get up from the floor. I prefer group-based exercise compared to individual exercise. Instructed exercise program is good, we will be discipline[d], and I will feel shame if [I am too] lazy to do it. From the beginning until now, there is more improvement in terms of visual setting for the virtual exercise program. The uploaded video makes [it] easier [for me] to follow compared to the live instructed program*
Participant’s response to the intervention	Challenges or difficulties	***P6:**** Small screen [on] my phone also [mean it is]not easy to me to follow the instruction. Now I get familiar with it, it is much easier* ***P2:**** The main challenge is internet connection distraction. Sometime, [the] line [is]not so good, connection was poor, the exercises suddenly stop, and I don’t know what to do next*
	Benefit	***P5:**** Last time I feel difficult to bend down and now is improving* ***P4:**** I feel my hand getting stronger, especially the left side. Besides that, this exercise helps my memories, because 10 years ago I [had a] stroke and I have poor coordination* ***P3:**** After performing the exercise, I sweat and I feel better, fresher*

The first theme was participants’ overall experience of the virtual exercise program using any device (mobile, tables, computer). Participants had limited previous experience of virtual program of any nature, with only two having previously participated in online classes (for religious purposes and music lessons). Participants with no prior experience in virtual exercise program agreed to trying an online exercise program to enable themselves to exercise at home safely during the COVID-19 pandemic. The second theme that emerged concerned participants’ perceptions of the virtual group exercise program design. Information on exercise intensity, tailoring to specific condition and design, as well as the delivery method, provided useful information for refining the program for the next phase. Most participants agreed that two sessions a week of virtual exercise were sufficient to suit their daily routines and that over an hour per session was ideal for them.

Lastly all participants mentioned that they were satisfied with the exercise design, as they perceived it to be a comprehensive program that involved multicomponent exercise. Most participants valued a virtual exercise program which used pre-recorded videos, compared to live demonstrations. Pre-recorded videos would involve recording exercise routines or demonstrations ahead of time, allowing participants to follow the content during the exercise session, while the researcher, acting as a physiotherapist, can monitor or guide participants. It was clearer and easier for participants to follow, while the instructor could monitor each participant’s movements and correct them when required.

The acceptability rates generally ranged from seven to nine with a median of nine. These participants were interested in joining the subsequent phase of the exercise program. The trial sessions recorded a 100% class attendance from the first to the second session with mean of 100%. The instructor observed high levels of engagement from participants in the class (with a mean score of 8/10), and participants were generally successful in understanding and replicating the instructions provided. They were able to perform the movements and exercises as demonstrated in the pre-recorded video, although there were occasional instances of incorrectly performing some of the movements.

### Effectiveness on outcome measures

**Fig. 1:**
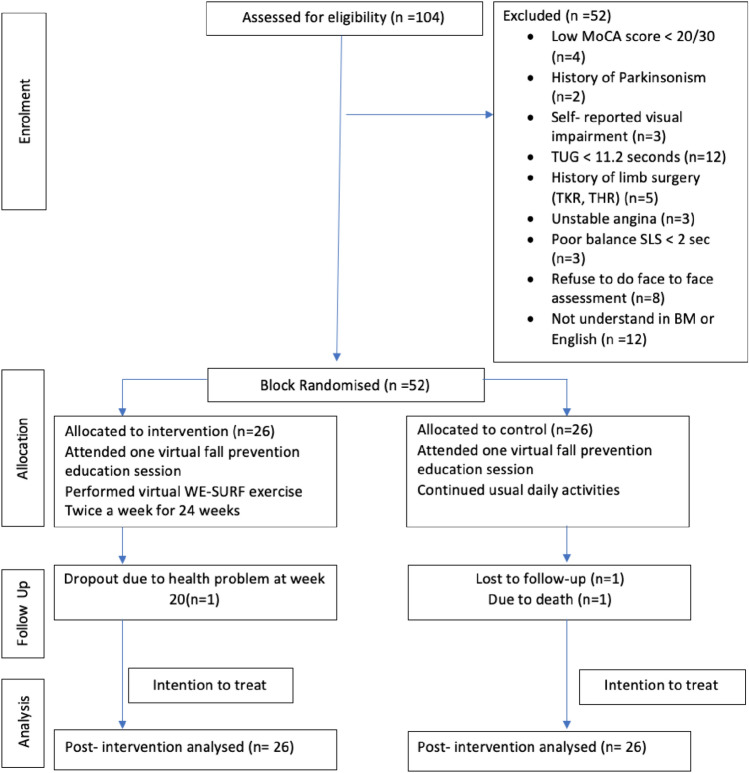
Consort diagram showing recruitment and retention of participants

After the preliminary study, screening continued, and 52 out of 104 older adults were found eligible to participate. Eight participants withdrew from the baseline assessment due to COVID-related reasons and refused to undergo face-to-face assessment. (Fig. 1).

Table 3 shows a total of 52 participants included in this study, randomised into the experimental group (WE-SURF™) (n = 26) and control group (n = 26). Majority of the participants were women (85%), Chinese (52%), unmarried/ windowed/divorced (71%) and with secondary education level (60%). Please note that three female participants (intervention: one; control: two) have a history of lower limb fracture within the past three to 10 years.

**Table 3:** Table for baseline characteristic for participants

Variables	Total N = 52	N (%)		P- value
		Experimental (n = 26)	Control (n = 26)	
Gender				
Male	8	2	6	0.25
Female	44	24	20	
**Age** (mean ± SD)	66.54 ± 5.15	66.96 ± 4.77	66.12 ± 5.56	0.56
Self- Efficacy Exercise				
(Mean ± SD)	58.44 ± 15.98	59.81 ± 14.51	57.07 ± 17.50	0.54
Ethnicity				
Malay	16	6	10	
Chinese	27	15	12	
Iban	6	5	1	0.14
Bidayuh	2	0	2	
Kayan	1	0	1	
Marital status				
Married	15	9	6	0.54
Unmarried/Windowed/Divorced	37	17	20	
Years of Education				
Secondary	31	17	14	
Diploma	12	5	7	0.43
Tertiary	9	4	5	
Working Status				
Working	4	2	2	0.70
No working	48	24	24	
Smoking History				
Smoker	1	0	1	0.50
Not smoking	51	26	25	
Alcohol History				
Yes	2	1	1	0.76
No	50	25	25	
History of fall				
Yes	21	10	11	0.29
No	31	16	15	
Living Status				
Alone	1	1	0	0.50
Not alone	51	25	26	

The results (Table 4) showed significant time x group interaction effects on risk of falls (TUG, p < 0.05), lower and upper limbs muscle strength (5STS & HGS) and balance (SLS). The experimental versus the control group showed greater improvements in the percentage mean change in SLS (111% vs 42%), 5STS (− 32% vs − 16%), TUG (− 27% vs − 17%) and dominant HGS (10% vs − 1%).

**Table 4:** Intervention effects on TUG, HGS, FTSTS, SLS, QOL, and FES tests from baseline to 24th weeks

Outcome Measures	Experimental (N = 26)	Control (N = 26)	GLM Repeated Measures		
			*p*-value	*p*-value	*p*-value
			Time effect (ηp^2^)	Group effect (ηp^2^)	Interaction Effect (ηp^2^)
TUG test (seconds)					
Baseline mean ± SD	12.53 ± 1.71	12.69 ± 1.94	< 0.01^**^	0.11	< 0.05^*^
24th week mean ± SD	9.17 ± 1.77	10.55 ± 2.54	(0.63)	(0.05)	(0.08)
5STS test (seconds)					
Baseline mean ± SD	15.63 ± 4.51	16.28 ± 3.64	< 0.01^**^	0.04^*^	0.03^*^
24th week mean ± SD	10.67 ± 2.59	13.61 ± 3.43	(0.53)	(0.08)	(0.09)
Dominant HGS (Kg)					
Baseline mean ± SD	21.58 ± 5.43	23.27 ± 7.22	0.10	0.79	0.03^*^
24th week mean ± SD	23.77 ± 4.63	22.96 ± 7.14	(0.05)	(0.01)	(0.09)
SLS (seconds)					
Baseline mean ± SD	18.29 ± 7.08	16.96 ± 5.03	< 0.01^**^	0.01 *	0.02^*^
24th week mean ± SD	38.70 ± 25.03	24.07 ± 14.71	(0.33)	(0.12)	(0.10)
FES-I(score)					
Baseline mean ± SD	16.77 ± 6.16	19.04 ± 9.35	< 0.01^**^	0.51	0.31
24th week mean ± SD	13.19 ± 6.96	13.58 ± 8.69	(0.33)	0.01	(0.02)
EQ-5D					
Baseline mean ± SD	76.15 ± 13.66	78.08 ± 6.65	0.05*	0.34	0.84
24th week mean ± SD	79.89 ± 12.49	82.65 ± 11.62	(0.08)	(0.02)	(0.01)

*FES-I* International Fall Efficacy Scale; *TUG* Time Up and Go test; *5STS* Five times sit to stand test; *HGS* Handgrip strength; *SLS* Single leg stance; *EQ-5D* Quality of life test; *GLM* General linear measures. ηp^2 –^ partial eta- squared

^*^p < 0.05

^**^p < 0.01

### Feasibility outcomes

The average of total attendance rate of the exercise program exceeded 81% (attended 39 out of 48 sessions). A total of 96% of the participants completed the study after 24 weeks. Eighty three percent of the participants recorded an absence for at least one session and the frequency of missed sessions ranged from one to eight. The common reasons for absence were COVID-19 vaccine appointments, resting after vaccine shot, personal commitments, and travelling. One of the adverse outcomes recorded was mild musculoskeletal related pain, experienced by nine older adults involving either the shoulder, ankle, low back and knee after the exercise program. Notably, all these participants had preexisting musculoskeletal pain disorders, such as ankle swelling, frozen shoulder, low back pain, and knee osteoarthritis. Moreover, upon further questioning, all the participants asserted that their musculoskeletal pain was not related to the virtual exercise and reported a reduction in pain upon completion compared to the initial session of the program.

The majority of participants scored 8/10 for exercise levels 1 and 2, while at level 3, it was 9/10, with a mean ranging from 7/10 to 9/10. Multiple interruptions occurred during virtual exercise sessions, including disturbances by pets (5 sessions), playful grandchildren (10 sessions), arguments with spouses (15 sessions), multitasking (7 sessions), visits to the toilet (15 sessions), and office work (12 sessions).

At the 6-month follow-up visit, four participants from the intervention group and three from the control group experienced at least one fall; however, it’s noteworthy that these falls did not occur during the exercise sessions. Two female participants from each group experienced mild musculoskeletal injuries (intervention: elbow and low back pain; control group: both had low back pain), lasting for a week due to falling, while three others had no complaints post-fall.

## Discussion

The WE- SURF™ exercise program appears to be acceptable and feasible among community-dwelling older adults with low and moderate risk of falls without any severe adverse events. While prior experience with virtual classes had been limited among older adults, this did not appear to limit their ability to participate in the virtual exercise program developed within this study. In a previous study conducted among pre-disabled older adults, good satisfaction level towards remote exercise program was reported [55]. Moreover, the COVID-19 pandemic underscored the importance of virtual exercise options, even among older adults who may not be considered technologically savvy [56, 57].

Safety of the older adults was the main concern in this study. Strict adherence to selection criteria was therefore practiced, whereby only those who were able to stand on one leg for more 10 s were included. As exercise program contained a balance challenge component, the prevention of a fall during exercise is vital to ensure continuation of the study and participant safety [58, 59]. Technology gadgets utilised by participants in this study consisted of the lowest range (low specification smartphone) to desktops with high processing speeds, which was similar to the findings from a previous study [60]. Smaller screen, low volume, and internet interruption were the common challenges reported in the preliminary test. However, these challenges are seldom reported in previous studies, probably because smartphones were used by many among the present study compared to other studies in the western countries [60, 61].

The high attendance and low dropout rates could be explained by the motivation techniques used in this study. A social media messaging group was created for each block of participant groups (total of 4 groups) and this led to a sense of belonging, connection, friendship among them with greetings, discussions about exercise, sharing of hobbies, and consultation about personal health conditions with the instructor. A similar technique has been reported previously [33]. In addition, the real time supervision and interaction between participants and instructor formed a dynamic interaction and an enjoyable atmosphere in the virtual environment.

Despite some musculoskeletal problems, participants were able to complete the program. It is noteworthy that the instructor, a physiotherapist with experience in geriatric physiotherapy played an active role in providing consultation regarding any problems encountered by the participants. For instance, self-management techniques with modifications to the exercise was provided to facilitate quick relief and continuous participation. Likewise, monitoring the perceived exertion level using Borg Rating of Perceived Exertion (RPE) scores from each participant during and after exercise sessions was advantageous in providing feedback from participants for exercise adaptations by the instructor. Intensity of exercises started with “light” (as in Borg Rating of perceived exertion level) and progressed to higher levels over the 3 month period. This could have led to higher engagement rates in level three compared to the first level of the exercise program as reported in previous related studies [62, 63]. Lastly, no fall or any other related injury occurred during the exercise session among participants in our present study.

The WE-SURF™ program was shown to be effective in improving balance and muscle strength. These findings suggest that similar results are possible even via virtual falls prevention exercises programs among older adults. This is expected as the program was designed based on evidence based fall prevention recommendations [19, 64]. WE-SURF™ fulfilled the requirements of exercise intensity of total 60 h, being multicomponent, combining challenging balance, resistance, aerobic, backward chaining and square stepping training [21, 65, 66]. In previous falls prevention programs that were conducted physically, it was demonstrated that there was an improvement in balance and muscle strength [67–70]. However, further studies are required to evaluate quality of life and fear of falls pertaining to virtual exercise programs. Longer interventions with inclusion of fear alleviating techniques may be required in such programs.

This study was limited by its execution during the COVID-19 pandemic which could have led to a higher acceptance and adherence rate resulting from the lack of any other competence engagement due to lock down and shielding which occurred among older adults. Further, while the exercise equipment such as a specially designed square-stepping mat, strap on weights and yoga mats were of low cost, it could limit its implementation especially among older adults from lower socio-economic groups. In addition, the requirement of an experienced physiotherapist as an instructor may not be possible for sustainability of such programs. However, knowledge transfer and echo training and step by step guide via digitalization are plausible for such programs to be maintained among older adults.

## Conclusion

In conclusion, WE-SURF™, a virtual supervised group-based fall prevention exercise program demonstrated acceptability, feasibility, and effectiveness among community-dwelling older adults with mild to moderate risk of falls, showing improvements in balance and lower limb muscle strength. Further research is needed to evaluate the implementation of the WE-SURF™ program as a fall’s prevention program among older adults, explore its long-term effects, and assess its impact on quality of life and fear of falling.

## Data Availability

All data available is shared in this paper.
